# Sleep Disorders and Fatigue among Patients with MS: The Role of Depression, Stress, and Anxiety

**DOI:** 10.1155/2024/6776758

**Published:** 2024-01-29

**Authors:** Nassim Zekibakhsh Mohammadi, Amir Sam Kianimoghadam, Niloofar Mikaeili, Seyedeh Samaneh Asgharian, Mahdieh Jafari, Abbas Masjedi-Arani

**Affiliations:** ^1^Department of Psychology, Faculty of Educational Sciences and Psychology, University of Mohaghegh Ardabili, Ardabil, Iran; ^2^Department of Clinical Psychology, Religion and Health Research Center, Shahid Beheshti University of Medical Sciences, Tehran, Iran; ^3^Department of Humanities, Ardabil Branch, Islamic Azad University, Ardabil, Iran; ^4^Department of Clinical Psychology, School of Medicine, Shahid Beheshti University of Medical Sciences, Tehran, Iran

## Abstract

Sleep disorders and fatigue represent prominent symptoms frequently experienced by individuals with multiple sclerosis (MS). Some psychological factors such as depression, stress, and anxiety seem to have a relationship with such problems. This study aimed to examine the role of depression, stress, and anxiety in predicting sleep disorders and fatigue among patients with MS. Employing a cross-sectional descriptive-correlational design, the study involved a sample size of 252 participants selected through purposive sampling based on inclusion and exclusion criteria. We utilized a demographic information questionnaire along with the Mini-Sleep Questionnaire (MSQ), Fatigue Severity Scale (FSS), and Depression, Anxiety, and Stress Scale (DASS-21) to collect data and analyzed them applying SPSS_22_, incorporating statistical measures including Pearson correlation and regression. The results of the Pearson correlation coefficient showed that sleep disorders had a positive and significant relationship with depression (*r* = 0.56; *P* < 0.001), stress (*r* = 0.40; *P* < 0.001), and anxiety (*r* = 0.52; *P* < 0.001). There was no significant relationship between age and the development of sleep disorders in total score (*r* = −0.001; *P* < 0.985), but age had a relationship with insomnia (*r* = −0.146; *P* < 0.021) and oversleeping (*r* = 0.153; *P* < 0.015). Age and fatigue did not have a significant relationship as well (*r* = −0.044; *P* < 0.941). In addition, fatigue had a positive and significant relationship with depression (*r* = 0.52; *P* < 0.001), stress (*r* = 0.48; *P* < 0.001), and anxiety (*r* = 0.54; *P* < 0.001). The results of the regression analysis also showed that depression, stress, and anxiety predict 0.37% of the total variance of sleep disorders (*F* = 48.34; *P* < 0.001) and 0.35% of the total variance of fatigue (*F* = 44.64; *P* < 0.001). Our findings suggest that depression, stress, and anxiety play a significant role in predicting sleep disorders and fatigue among patients with MS. This study has been reported in accordance with the TREND checklist for nonrandomized trials.

## 1. Introduction

2.5 million individuals globally [1], with 57 in 100,000 in Iran [2], are involved with multiple sclerosis (MS). MS, a chronic [3] neurodegenerative disease of the central nervous system (CNS) [4], is marked by the demyelination of axons. This demyelination hinders the effective transmission of information between various brain regions [5], causing both physical and psychological challenges [6]. Symptoms encompass sensory, visual, motor, and cognitive impairments, accompanied by fatigue [5, 7, 8] and mood disorders [9].

A substantial proportion of individuals with MS suffer from sleep disturbances [10, 11]. These issues encompass difficulties in falling asleep, staying awake at night, waking up excessively early, and struggling to return to sleep [12]. Patients with MS commonly report higher levels of daytime sleepiness and sleep disruptions compared to the general population [13]. Numerous studies indicate varying percentages of sleep disorders among patients with MS [7, 12, 14] in different percentages as follows: 50% [15], 60% [16], and 70% [17]. These disturbances contribute to bothersome consequences, including heightened daytime sleepiness, impaired memory and learning, diminished concentration, mood fluctuations, and increased fatigue [12, 18].

Sleep difficulties are closely linked to weakened mental health [19, 20]. Depression is one of the most prevalent psychiatric disorders in up to 50% of patients with MS [21, 22], resulting in poor sleep quality [23], sleep difficulties [20, 24], and sleep disturbance, insomnia in particular [12, 25]. Symptoms of depression, like anhedonia and reduced physical activity, may lead to increased time spent sleeping, but the sleep is often less efficient, restful, and uninterrupted [26]. Stress and anxiety, other common psychologic comorbidities in MS [27, 28], have a heightened risk of manifestation compared to the general population [29]. There exists a reciprocal relationship between stress and sleep issues [30, 31], where psychosocial stress exacerbates sleep disorders [32]. Anxiety, strongly associated with sleep disruptions [33–35], contributes to insomnia among patients with MS as well [36]. The literature underscores substantial connections between sleep disorders in patients with MS and mental health issues, including depression, stress, and anxiety.

Fatigue stands out as another prevalent and incapacitating symptom in patients with MS [37, 38] with a prevalence ranging from 50% to 83% [38–41]. It refers to a lack of physical and/or mental energy perceived as a weakness or lassitude interfering with daily activities among patients with MS [13, 42]. Evidence indicates associations between fatigue and lesions in the frontal and temporal regions [43–45] as well as indirect psychological factors impacting various aspects of patients' lives [46]. Fatigue can significantly affect quality of life [47], employment, social engagement, family life, and both physical and psychological function [48]. Common psychological issues linked to fatigue in MS patients include depression, anxiety [49], and stress [50]. Depression particularly influences mental fatigue [51], with research revealing a 3.6-fold increase in fatigue among depressed patients [52]. The use of stimulants and antidepressants has demonstrated a significant reduction in fatigue symptoms, emphasizing this strong connection [52, 53]. Fatigue can also be triggered by previously perceived stress [54, 55], and chronic stress is likely to predict increased fatigue [50]. Based on the evidence, anxiety is another psychological factor associated with higher levels of fatigue [28, 49, 56]. In summary, research suggests a compelling relationship between fatigue and psychological issues such as depression, stress, and anxiety.

The existing literature in the field of MS underscores the substantial prevalence of sleep disturbances [57] and fatigue [39] among patients, with a significant correlation to psychological issues like depression, stress, and anxiety [22, 29]. To enhance the efficacy of therapies for sleep disorders and fatigue among patients with MS, scholars require more detailed data on the contributing factors (in this study, including depression, stress, and anxiety) in developing those problems. While the relationship between these variables and sleep disorders and fatigue is established, there is a gap in research regarding the specific roles of depression, stress, and anxiety in the development and persistence of these issues among patients with MS. This study aimed to address this gap by examining the predictive role of depression, stress, and anxiety in sleep disorders and fatigue among patients with MS. The goal was to provide more reliable data for future studies, enabling scholars to develop more effective therapies for sleep disorders and fatigue in this population.

## 2. Materials and Methods

### 2.1. Procedures and Participants

This research adopted a cross-sectional descriptive-correlational design, selecting depression, stress, and anxiety as predictive variables to predict sleep disorders and fatigue among patients with MS. Following ethical approval from the Medical University of Shahid Beheshti and confirmation from the Iranian MS Association, in order to form the study sample, we calculated the sample size using Free Statistics Calculators software, version 4. Considering the effect size of 0.06, power of 0.9, predictor variables of 3, and alpha of 0.5, the sample size was determined to be 233 patients, and because of neutralizing the sample drop effects, we finalized the sample size with 254 participants [58, 59]. In order to collect data, first, we called an online invitation through the Iranian MS Association to its members in May, 2022. Then, we applied the purposive sampling method through inclusion and exclusion criteria to form the study sample. The inclusion criteria were having the diagnosis of multiple sclerosis and being in the age range of 18–65, and the exclusion criteria were the participants' fatigue and their unwillingness to continue. The Porsline online platform facilitated questionnaire distribution, ensuring participants' understanding of the study objectives and obtaining their consent. Ultimately, 254 association members completed the questionnaires remotely over two weeks. Demographic features of participants are detailed in Table 1.

## 3. Measures

### 3.1. Mini-Sleep Questionnaire (MSQ)

The Mini-Sleep Questionnaire (MSQ) is a self-report scale designed to assess current sleep quality, incorporating six questions regarding the frequency of sleep difficulties. It aims to evaluate various dimensions of sleep disorders, including insomnia and oversleeping, utilizing a five-point Likert scale. The total score is derived by summing the scores of each question. Manavipour's research confirmed the content validity of the questionnaire through exploratory factor analysis. The reliability, assessed using Cronbach's alpha, was reported at 0.79, indicating a satisfactory level of internal consistency [60].

### 3.2. Fatigue Severity Scale (FSS)

The fatigue severity scale developed by Krupp et al. [61] serves to gauge the intensity of fatigue in individuals with multiple sclerosis and lupus. Psychometric examination ensured its reliability and validity. Comprising nine items derived from a 28-item fatigue questionnaire, it assesses a person's subjective perception of fatigue on a seven-point Likert scale (1–7). Scores range from 9 to 63, with scores above 45 indicating a high level of fatigue. Krupp et al. [61] and Azimian et al. [62] reported Cronbach's alpha values of 0.88 and 0.96, respectively, attesting to the scale's robust internal consistency.

### 3.3. Depression, Anxiety, and Stress Scale (DASS-21)

The Depression, Anxiety, and Stress Scale was developed by Lovibond and Lovibond in 1995 to measure depression, anxiety, and stress through 21 statements and is rated on a four-point Likert scale (0–3). Lovibond reported a validity of 0.77 for DASS-21 [63]. In addition, Fathi-Ashtiani and Dastani [64] affirmed the reliability of the depression, anxiety, stress, and depression-anxiety-stress subscales with values of 0.89, 0.84, 0.82, and 0.83, respectively [64]. These findings support the robustness of the scale in measuring psychological constructs effectively.

### 3.4. Analytic Plan

We employed the exclude cases listwise method to eliminate missing data (0.80%) and applied SPSS_22_ software to analyze the remaining 252 questionnaires data. In this regard, we performed descriptive statistics measures including Mean and Standard Deviation to provide an overview of the data. In addition, we utilized inferential statistics measures including Pearson correlation and regression to explore relationships and predict outcomes within the dataset.

## 4. Results and Discussion

### 4.1. Results

Table 2 shows the results of descriptive statistics of research variables including depression, stress, anxiety, sleep disorders, and fatigue and the Pearson correlation coefficient among them. According to the results, the mean of depression, stress, anxiety, sleep disorders, and fatigue was 11.51, 12.24, 8.27, 16.90, and 43.64, respectively, and their standard deviation was 6.04, 5.33, 5.47, 5.30, and 14.14, respectively.

In order to check the normality of the data, we calculated the values of skewness and kurtosis of the variables and found out that these values for all variables (depression, stress, anxiety, sleep disorders, and fatigue) were between −1 and +1. Therefore, the normality of the data was confirmed [65]. We calculated the value of the reliability coefficient of the variables by Cronbach's alpha measure and reported the values of 0.80, 0.88, 0.85, 073, and 0.93 for depression, stress, anxiety, sleep disorders, and fatigue, respectively. The results of the Durbin–Watson statistic, variance tolerance index (VIF), and Tolerance showed that the predictor variables were independent of each other, and multicollinearity was not seen. In order to examine the relationship between the predictor variables (depression, stress, and anxiety) and the sleep disorders and fatigue, we used Pearson correlation coefficient and regression measures. According to the results of Pearson correlation coefficient in Table 2, sleep disorders have a positive and significant relationship with depression (*r* = 0.56; *P* < 0.001), stress (*r* = 0.40; *P* < 0.001), and anxiety (*r* = 0.52; *P* < 0.001). Likewise, there is a positive and significant relationship between fatigue and depression (*r* = 0.52; *P* < 0.001), stress (*r* = 0.48; *P* < 0.001), and anxiety (*r* = 0.54; *P* < 0.001). In addition, we found a relationship between age and insomnia (*r* = −0.146; *P* < 0.021) and oversleeping (*r* = 0.153; *P* < 0.015); however, there is no significant relationship between age and the development of sleep disorders in total score (*r* = −0.001; *P* < 0.985) and between age and fatigue (*r* = −0.044; *P* < 0.941).

The results of the regression analysis showed that the predictive variables including depression, stress, and anxiety predict 0.37% of the total variance of sleep disorders, and the ANOVA measure results showed the significance of regression (*F* = 48.34; *P* < 0.001). The results of the regression analysis for fatigue indicated that the predictive variables predict 0.35% of the total variance of this variable, and the significance of regression for this variable was also confirmed by the ANOVA measure (*F* = 44.64; *P* < 0.001). These results are shown in Table 3.

## 5. Discussion

In line with the existing literature indicating that approximately half of patients with MS experience sleep disorders and about 90% contend with fatigue [12, 66], this study aimed to discern the contributory factors to these prevalent symptoms. The investigation focused on evaluating the role of depression, stress, and anxiety in predicting sleep disorders and fatigue among patients with MS. The findings underscored significant relationships between the dependent variables (sleep disorders and fatigue) and the predictive variables (depression, stress, and anxiety). Importantly, these variables demonstrated the ability to predict a proportion of sleep disorders and fatigue among patients with MS.

The relationship between sleep disorders and depression in the general population has been shown in previous studies as well [25, 67]. The research studies of Lamis et al. [24] and Zhang et al. [12] have also shown such a relationship among patients with MS. To explain these results, it can be referred to the common genetic and neurobiological roots of sleep disorders and depression. It is shown in studies that there is a responsible gene for both of these unhealthy situations and individuals having that specific gene are susceptible to depression and sleep difficulties simultaneously [68]. The studies have also demonstrated the role of the hypothalamo-pituitary-adrenal (HPA) axis in sleep disorders and depression [69] which is another common root for them. In addition, functional connectivity in the brain mediates the association between depressive problems and sleep quality [70]. Beyond neurobiology, nonneurobiological factors such as the side effects of depression, including anhedonia and physical inactivity, contribute to increased time spent sleeping without achieving efficient, restful, and uninterrupted sleep [26]. Together, the brain connectivity, common genetic and neurobiological roots for sleep disorders and depression, and the mentioned nonneurobiological reason provide insights into the intricate relationship between sleep disorders and depression among patients with MS.

The study's findings also revealed a significant relationship between sleep disorders and stress, consistent with prior research indicating that stress can contribute to sleep difficulties [30, 31, 71]. According to the past research study and from the physiological perspective, the HPA axis is also responsible for the close relationship between sleep disorders and stress [72, 73]. This happens through decreasing slow waves and rapid eye movement (REM) and sleep deprivation caused by stress [73]. This mechanism illustrates the positive relationship between sleep disorders and stress.

Moreover, the investigation identified a connection between sleep disorders and anxiety. Some past studies have revealed such results as well [33, 36, 74]. Some studies have shown that anxiety is among the most significant psychological contributory factors to patients with MS's sleep difficulties [34, 75]. Shared genetic and neurological factors responsible for both anxiety and sleep disorders have been identified in several studies [76–78]. In addition, the role of neurotransmitters such as 5-hydroxytryptamine (5-HT) and norepinephrine (NE) in the manifestation of both anxiety and sleep disorders has been proven [79]. Studies supporting the common neurobiological roots of anxiety and sleep disorders, along with improvements in MS patients' sleep quality following anxiety treatment [80], affirm the results of both past and present research on this relationship.

The results of this study also showed that psychological problems including depression, stress, and anxiety in patients with MS can contribute to predict sleep disorders by 37 percent. While no prior study precisely mirrors these results, the existing literature on the connections among these variables, coupled with shared physiological and genetic origins for sleep difficulties and psychological problems [30, 68, 69, 72], offers a comprehensive explanation for the role of the predictor variables including depression, stress, and anxiety in forecasting sleep disorders among patients with MS.

Regarding the association between fatigue and depression, the study's findings align with some prior research [38, 52, 81]. A plausible explanation emerges from the neurophysiological perspective, suggesting that fatigue and depression in patients with MS share potential origins. This perspective emphasizes that both fatigue and depression among such patients have a strong relationship with smaller cortical surface area and volumes on brain MRI [82], along with activated immune-inflammatory pathways [83]. These findings elucidate the robust relationship between fatigue and depression in this particular population.

The results also highlight a connection between fatigue and stress, a relationship consistent with findings from other studies [50, 71, 84]. Nag et al., in research related to applying stress-reducing activities for patients with MS, demonstrated that reducing stress for these patients correlated with fewer reports of fatigue [85]. This could be attributed to chronic stress depleting the body's resources, resulting in diminished physical energy and a prolonged sense of fatigue. Conversely, lower stress levels may preserve the body's energy and resources, contributing to a reduced sense of fatigue. This mechanism offers an explanation for the observed relationship between stress and fatigue.

Furthermore, this study also established a significant relationship between fatigue and anxiety. Some past studies have also demonstrated consistent results regarding this relationship [28, 38, 49]. In explaining such a relationship, Wilson et al. illustrate that anxiety results in the arousal of the sympathetic nervous system. As this arousal continues, the rate of adrenal and cortisol increases in the blood and leads to “adrenal fatigue”—a prolonged feeling of fatigue associated with anxiety [86]. The mediating role of the sympathetic nervous system in the connection between anxiety and fatigue elucidates the significant relationship between these two variables.

We also found out that 35 percent of the variance of fatigue can be accounted for by depression, stress, and anxiety. Past studies have revealed similar findings for other communities [50, 87, 88]. However, to our best knowledge, there has not been research on these variables' role in predicting fatigue among patients with MS. The mentioned studies above explain the role of depression in predicting fatigue through common impaired brain regions—smaller cortical surface area and volumes on brain—along with activated immune-inflammatory pathways. Based on of the described research, stress and anxiety predict fatigue through the HPA axis and arousal of the sympathetic nervous system. Collectively, these research findings point to shared neurobiological foundations among psychological issues in patients with MS, such as depression, stress, anxiety, and fatigue, offering insight into how the predictive variables contribute to fatigue.

## 6. Conclusion

The study results establish a significant and positive relationship between predictor variables, including depression, stress, and anxiety, and dependent variables, namely, sleep disorders and fatigue, among patients with MS. Notably, the predictor variables account for 37 percent of the variance in sleep disorders and 35 percent in fatigue among this population. These findings highlight the importance of healthcare professionals addressing the contributory factors to sleep disorders and fatigue in patients with MS, with particular emphasis on depression, stress, and anxiety, as investigated in this study. In conclusion, reducing levels of depression, stress, and anxiety is suggested to lead to decreased occurrences of sleep disorders and fatigue among patients with MS. This, in turn, has the potential to alleviate the negative side symptoms of MS, enhance positive experiences, and improve the overall quality of life for these individuals.

## Figures and Tables

**Table 1:** Demographic features of participants.

Demographic features	Percentage
Sex	
Male	18.3
Female	81.7
Age	
18–39	75
40–65	25
Marital status	
Single	35.3
Married	64.7
Job status	
Employed	40.9
Unemployed	59.1
Education status	
Under diploma	4
Diploma	20.6
Associate degree	6.3
Bachelor's degree	47.6
Master's degree and above	21.4
Children number	
Zero	53.2
One	26.6
Two	17.5
Three	2.4
+ four	0.4

**Table 2:** Descriptive statistics and the Pearson correlation coefficient values for the variables.

Variables	Mean	SD	1	2	3	4
(1) Depression	11.51	6.04				
(2) Stress	12.24	5.33	0.65^*∗∗*^			
(3) Anxiety	8.27	5.47	0.62^*∗∗*^	0.68^*∗∗*^		
(4) Sleep disorders	16.90	5.30	0.56^*∗∗*^	0.40^*∗∗*^	0.52^*∗∗*^	
(5) Fatigue	43.64	14.14	0.52^*∗∗*^	0.48^*∗∗*^	0.54^*∗∗*^	0.44^*∗∗*^

^*∗∗*^ *P* < 0.01.

**Table 3:** The regression measure results for dependent variables based on depression, stress, and anxiety.

Dependent variable	Predictive variables	*R* ^2^	*F*	Sig: *F*	*B*	SEB	Beta	*t*	Sig
Sleep disorders		0.37	48.34	0.00					
	Depression				0.39	0.06	0.43	6.09	0.00
	Stress				−0.11	0.07	−0.10	−1.42	0.16
	Anxiety				0.33	0.07	0.33	4.55	0.00

Fatigue		0.35	44.64	0.00					
	Depression				0.62	0.17	0.27	3.74	0.00
	Stress				0.28	0.20	0.10	1.37	0.17
	Anxiety				0.78	0.19	0.30	4.1	0.00

## Data Availability

Due to patient privacy consideration, the authors are not allowed to publicly publish the research data.

## Additional Points

*Limitations*. This study is subject to certain limitations. The use of self-reported questionnaires introduces potential biases from participants. The restriction of participants to adults within the age range of 18–65 may limit the generalizability of the results. A large difference in the sample composition between male and female participants could potentially impact the study's outcomes. In addition, the employed analytical plan prevents the establishment of cause-effect relationships, as the findings are confined to correlational effects. To enhance the accuracy of data, future research may consider incorporating interviews for variable measurement. Broadening the age range and achieving a more balanced gender ratio among participants would also enhance the generalizability of the results.
